# Real-world utilization pattern of dydrogesterone in 7287 Indian women with obstetric and gynecological conditions: data from multicentric, retrospective study

**DOI:** 10.61622/rbgo/2024AO18

**Published:** 2024-03-15

**Authors:** Jaydeep Tank, Sanjay Gupte, Purna Chandra Mahapatra, Jayanthi Reddy, Pratima Mittal, Ashish Kumar Mukhopadhyay, Lila Vyas, Achla Batra, Mahesh Gupta, Sunita Tandulwadkar, Sunita Chandra, Vidya Bhat, Kawita Bapat, Parikshit Tank, Ketan Kulkarni, Onkar Swami

**Affiliations:** 1 Ashwini Maternity and Surgical Hospital Mumbai India Ashwini Maternity and Surgical Hospital, Mumbai, India.; 2 Gupte Hospital and Centre for Research in Reproduction Pune - Obstetrics and Gynecology Pune Maharashtra India Gupte Hospital and Centre for Research in Reproduction, Pune - Obstetrics and Gynecology, Pune, Maharashtra, India.; 3 Prachee Nursing Home Cuttack India Prachee Nursing Home, Cuttack, India.; 4 J. J. Hospital Hyderabad India J. J. Hospital, Hyderabad, Hyderabad, India.; 5 Vardhman Mahavir Medical College and Safdarjung Hospital New Delhi India Vardhman Mahavir Medical College and Safdarjung Hospital, New Delhi, India.; 6 CSS College of Obstetrics Kolkata India CSS College of Obstetrics, Gynae. & Child health, Kolkata, India.; 7 Vyas Clinic Jaipur Rajasthan India Vyas Clinic, Jaipur, Rajasthan Jaipur, India.; 8 Pushpam Hospital Ahmedabad India Pushpam Hospital, Ahmedabad, India.; 9 Ruby Hall Clinic IVF and Endoscopy Centre Pune India IVF and Endoscopy Centre, Ruby Hall Clinic, Pune, India.; 10 Rajendra Nagar Hospital and IVF Center Lucknow India Rajendra Nagar Hospital and IVF Center, Lucknow, India.; 11 Radhakrishna Multispecialty Hospital Bangalore Karnataka India Radhakrishna Multispecialty Hospital, Bangalore, Karnataka, India.; 12 Bapat Hospital Indore Madhya Pradesh India Bapat Hospital, Indore, Madhya Pradesh, India, Indore, India.; 13 Emcure Pharmaceuticals Pune Maharashtra India Emcure Pharmaceuticals, Pune, Maharashtra, India.

**Keywords:** Comorbid conditions, Concomitant medications, Dydrogesterone, Gynecological conditions, Indian women, Risk factors, Threatened abortion, Utilization pattern

## Abstract

**Objective:**

Despite the literature on dydrogesterone, studies on dydrogesterone utilization patterns are largely lacking in Indian patients.

**Methods:**

This was a multi-center, retrospective, observational, cross-sectional, and descriptive study across 817 centers in India. Data of patients who received dydrogesterone in past and provided consent for future use of their medical record for research purpose was were retrieved and analyzed.

**Results:**

Data of 7287 subjects (aged 29.55±4.84 years) was analyzed. Threatened abortion was the most common indication for which the subjects received dydrogesterone (46.9%) followed by recurrent pregnancy loss. Polycystic ovary syndrome (PCOS), thyroid disorders and anemia were the most common comorbid conditions and prior pregnancy loss, advanced maternal age and obesity were the most common risk factors seen in subjects who received dydrogesterone. Total 27.5% of subjects received a loading dose of dydrogesterone, and majority (64%) received 40 mg as loading dose. 10 mg dose was used as maintenance or regular dose in 81.4% of the subjects. Twice daily (BID) was the most common dosing frequency (66.6%). The most common concomitant medications being taken by the subjects on dydrogesterone included folic acid (45.1%), iron supplements (30.3%) and calcium and vitamin D3 supplements (25.5%). Another progesterone preparation (oral, injection, vaginal, tubal) other than dydrogesterone was used concurrently in 7.8% of subjects.

**Conclusion:**

The study helped to identify the patient population that is benefitted by dydrogesterone and the preferred indications, risk factors, comorbid conditions and concomitant medication used in this patient population at real-life scenario.

## Introduction

Progesterone has an important place in the management of obstetric and gynecological conditions.^(1-3)^ Progesterone can be given orally, intramuscularly, subcutaneously, intravaginally and through rectal routes.^(4)^ Of these, oral route is most convenient for patients.^(4)^ However, oral micronized progesterone has limited use in clinical practice because it undergoes extensive first-pass metabolism and therefore has relatively low bioavailability.^(4)^

Dydrogesterone is a stereoisomer of progesterone that is pharmacologically close to endogenous progesterone.^(1,5)^ It has high oral bioavailability, is more specific for progesterone receptors than micronized progesterone, and therefore a 10–20 times lower oral dose than micronized progesterone is required to exert the pharmacological action.^(1,4,5)^ The androgenic, glucocorticoid, mineralcorticoid or estrogenic side-effects of dydrogesterone are significantly less compared with micronized progesterone.^(1,6)^

Dydrogesterone has been in use for over 60 years, and has demonstrated a favorable efficacy, safety and tolerability profile across multiple obstetric and gynecological indications.^(1,7-9)^ Dydrogesterone has been efficiently used for progesterone-deficient obstetrical conditions such as threatened abortion, recurrent pregnancy loss, infertility due to luteal phase deficit, and gynecological conditions such as endometriosis, dysfunctional uterine bleeding, secondary amenorrhea, irregular menstrual cycles and premenstrual syndrome.^(1,4,7,10-19)^ Dydrogesterone is the most commonly prescribed progesterone during pregnancy and for threatened abortion.^(9,20)^ Oral dydrogesterone carries the least risk of miscarriage while treating threatened abortion and recurrent pregnancy loss as compared to other progesterone.^(7,13)^

It has been found to be as effective as micronized progesterone for luteal phase support during in-vitro fertilization (IVF) cycles and for treating threatened miscarriages due to corpus luteum insufficiency.^(10-12)^ The use of dydrogesterone has been correlated with higher pregnancy rate and live birth rate than with micronized progesterone in patients requiring luteal phase support during IVF.^(4)^

Despite the extensive literature on dydrogesterone, studies on dydrogesterone utilization patterns are largely lacking in Indian patients. Therefore, the present study was conducted with objective of assessing the dydrogesterone utilization patterns in various obstetric and gynecological conditions in Indian women at real life scenario. The study also aimed to identify the frequencies of risk factors and co-morbidities in patients receiving dydrogesterone and the concomitant medications.

## Methods

This was a multi-center, retrospective, observational, cross-sectional, and descriptive study conducted across 817 centers spanning major urban and rural regions of India. A case report form (CRF) was designed prior to the study and shared with the Obstetricians and Gynecologists across India. Each center was given 10 CRFs. data was filled from the medical records of women ≥ 18 years of age who received dydrogesterone in the past as their standard of care (SOC) for any obstetric or gynecologic condition.

The study subjects received oral dydrogesterone in a dosage considered appropriate by their physician for that condition. The locally approved prescribing information for dydrogesterone is provided in chart 1.^(21)^

**Chart 1:** Dydrogesterone prescribing information

Indication	Dose
Threatened abortion	An initial loading dose of up to 40 mg may be given followed by 20 or 30 mg per day until symptoms remit
Recurrent pregnancy loss	10 mg BID until the twentieth week of pregnancy
Infertility due to luteal insufficiency	10 or 20 mg daily starting with the second half of the menstrual cycle until the first day of the next cycle for three consecutive cycles
Endometriosis	10 to 30 mg daily from day 5 to day 25 of the cycle or continuously
Irregular cycles	10 or 20 mg daily starting with the second half of the menstrual cycle until the first day of the next cycle
Pre-menstrual syndrome	10 mg daily starting with the second half of the menstrual cycle until the first day of the next cycle
Dysfunctional uterine bleeding	When treatment is started to arrest a bleeding episode, 20 or 30 mg dydrogesterone per day is to be given for up to 10 days. For continuous treatment, 10 or 20 mg dydrogesterone per day should be given during the second half of the menstrual cycle.
Secondary amenorrhea	10 or 20 mg dydrogesterone per day, to be given daily for 14 days during the second half of the theoretical menstrual cycle to produce an optimum secretory transformation of an endometrium that has been adequately primed with either endogenous or exogenous estrogen
Hormone replacement therapy	– Continuous sequential therapy: An estrogen is dosed continuously and one tablet of 10 mg dydrogesterone is added for the last 14 days of every 28 day cycle, in a sequential manner. – Cyclic therapy: When an estrogen is dosed cyclically with a treatment-free interval, usually 21 days on and 7 days off. One tablet of 10 mg dydrogesterone is added for the last 12 -14 days of estrogen therapy. – Depending on the clinical response, the dosage can subsequently be adjusted to 20 mg dydrogesterone per day.

The following data was retrieved from medical records and filled in the CRFs: demographic details, comorbidities, risk factors, indication, dose, dosing frequency, regimen and duration of treatment of dydrogesterone in various obstetric and gynecological conditions and concomitant treatment used along with the dydrogesterone. All the data from the 817 centers was received at a central facility of the Sponsor, checked for inconsistencies, and entered in Microsoft Excel sheet. The data was analyzed using descriptive statistical methods. Categorical data was represented as frequencies and percentages, while quantitative data was described as mean ± standard deviation (SD) or median with interquartile range (IQR) as appropriate. Given the retrospective nature of the study, informed constent could not be taken from the patients whose data was collected from the medical records. However, the study was conducted according to the Principles of Helsinki. Further, this study was approved by the Deccan Independent Ethics Committee.

## Results

### Baseline characteristics

**Figure 1:**
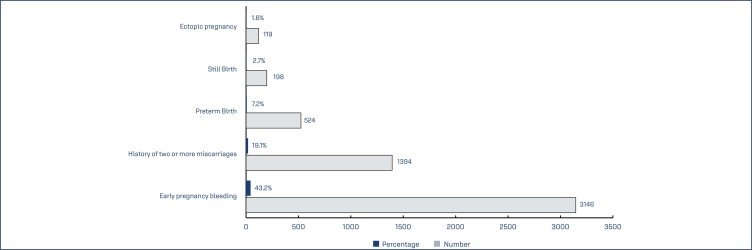
Causes of previous pregnancy loss

Data of 7287 subjects (aged 29.55±4.84 years) who had received dydrogesterone as part of their SOC were included in the study; Demographic details of the subjects, and their obstetric history are captured in (Table 1). Of the subjects, 83.3% were pregnant. A total of 73.8% of subjects had a previous pregnancy loss, and 43.2% of these had experienced early pregnancy bleeding during the previous miscarriage. Other causes of previous pregnancy loss are depicted in (Figure 1).

**Table 1:** Demographic profile of study subjects

	n	MEAN±SD	MEDIAN (IQR)	RANGE (Min-Max)
Age (year)	7108	29.55±4.84	29(26,32)	18 to 45
Weight (kg)	7062	59.38±9.35	59(53,65)	38 to 113
Height (cm)	7072	156.79±7.2	156(152,161)	136 to 179
BMI (KG/m^2^)	7013	24.23±3.84	23.83(21.76,26.11)	12.91 to 47.11
Gravida	3770	-	2(1,3)	0 to 10
Para	3265	-	0(0,1)	0 to 6
Abortions	3273	-	1(0,1)	0 to 7
Living offsprings	2712	-	0(0,1)	0 to 3

### Frequency of comorbid conditions in subjects receiving dydrogesterone

**Figure 2:**
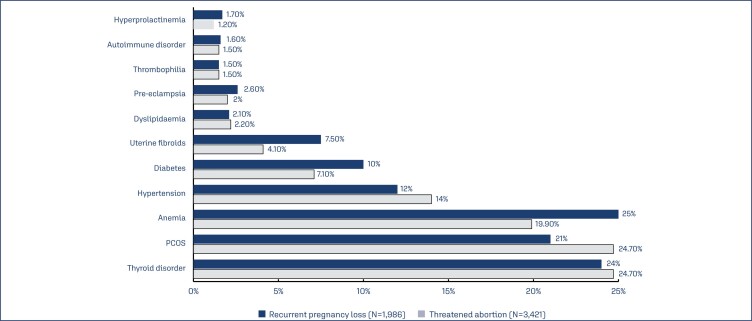
Common comorbidities in subjects with threatened abortion and recurrent pregnancy loss

Polycystic ovary syndrome (PCOS) and thyroid disorders were the most common comorbid conditions identified in 23% and 21% of subjects, respectively. Other comorbid conditions identified (Table 2) were anemia and hypertension in 19.9% and 13.3% of subjects, respectively. PCOS (24.7% and 21%), thyroid disorders (24.7% and 24%) and anemia (19.9% and 25%) were the most common comorbid conditions seen in subjects who received dydrogesterone for threatened abortion and recurrent pregnancy loss, respectively. PCOS was the most common comorbid condition in subjects with threatened abortion and anemia was the most common comorbid conditions in subjects with recurrent pregnancy loss. Other comorbid conditions seen in subjects with these two conditions are depicted in (Figure 2).

**Table 2:** Risk factors and comorbid conditions identified in subjects prescribed dydrogesterone

n=7287	n(%)
**Risk factors**	
Prior pregnancy loss	1981(27.2)
Advanced maternal age	1397(19.2)
Obesity	1177(16.2)
Family History	761(10.4)
Advanced paternal age	356(4.9)
Associated medical condition	322(4.4)
Alcohol Consumption	176(2.4)
Defects in the uterine and fallopian tube	175(2.4)
Smoking	164(2.3)
**Comorbid conditions**	
PCOS	1678(23)
Thyroid Disorder	1528(21)
Anaemia	1448(19.9)
Hypertension	971(13.3)
Diabetes	545(7.5)
Uterine fibroids	287(3.9)
Dyslipidaemia	191(2.6)
Pre-eclampsia	141(1.9)
Thrombophilia	138(1.9)
Autoimmune disorder	132(1.8)
Hyperprolactinemia	95(1.3)

### Frequency of risk factors identified in subjects receiving dydrogesterone

**Figure 3:**
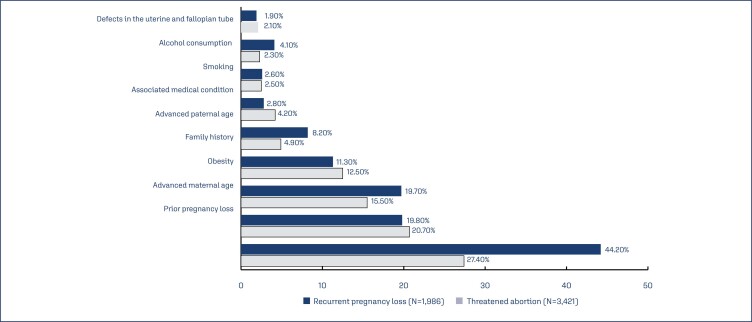
Common risk factors in subjects with threatened abortion and recurrent pregnancy loss

Prior pregnancy loss was the most common risk factor identified in 27.2% of subjects who received dydrogesterone. Other risk factors identified (Table 2) were advanced maternal age (19.2%) and obesity (16.2%). Prior pregnancy loss (27.4% and 44.2%), advanced maternal age (20.7% and 19.8%) and obesity (15.5% and 19.7%) were the most common risk factors seen in subjects who received dydrogesterone for threatened abortion and recurrent pregnancy loss, respectively. Other risk factors seen in subjects with these two conditions are depicted in (Figure 3).

### Dydrogesterone utilization pattern

#### Indications for dydrogesterone use

Dydrogesterone was most commonly prescribed for obstretrical conditions. Threatened abortion was the most common indication for which the subjects received dydrogesterone (46.9%) followed by recurrent pregnancy loss (27.3%). Dydrohesterone was also prescribed for several gynecological conditions. The most common gynecological condition for which subjects received dydrogesterone was infertility due to luteal phase insufficiency. Other gynecological indications for which the subjects received dydrogesterone are mentioned in (Table 3).

**Table 3:** Dydrogesterone utilization pattern: indications, frequency, dosage, dosing regime (timing)

Indications for dydrogesterone	
n=7287	n(%)
Obstetric conditions	
Threatened abortion	3421(46.9)
Recurrent pregnancy loss	1986(27.3)
Gynecological causes	
Infertility due to luteal phase insufficiency	843(11.6)
Luteal phase support as a part of ART treatment	750(10.3)
Dysfunctional uterine bleeding	235(3.2)
Endometriosis	208(2.9)
Secondary Amenorrhea	79(1.1)
Hormone replacement therapy	74(1)
Loading dose, dosing frequency, dosing regime (timing)	
n=7287	n(%)
Loading dose prescribed	2007(27.5)
No loading dose prescribed	5280(72.5)
Loading dose prescribed (n=2007)	
20 mg	387(19.3)
30 mg	318(15.8)
40 mg	1285(64.0)
50 mg	3(0.1)%
60 mg	13(0.6)%
80 mg	1(0.0)%
Maintenance dose/starting without loading dose (n=5280)	
10 mg	4299(81.4)
20 mg	770(14.6)
30 mg	134(2.5)
40 mg	72(1.4)
50 mg	4(0.08)
60 mg	1(0.02)
Dosing frequency (n=6851)	
OD	869(12.7)
BID	4562(66.6)
TID	1420(20.7)
Dosing regimen (n=7286)	
Morning	5744(78.8)
Afternoon	1381(19)
Evening	5706(78.3)
Cyclical/continuous (n=4791)	
Cyclical Treatment	1072(22.4)
Continuous Treatment	3719(77.6)

#### Dosage, dosing and regime

**Figure 4:**
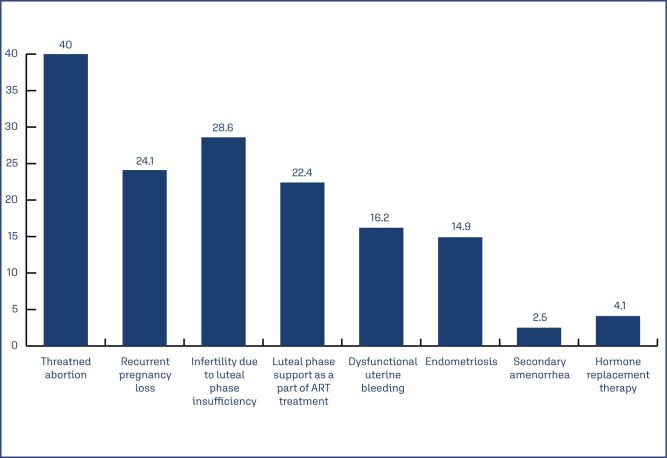
Proportion of cases by indication who received dydrogesterone loading dose

Slightly more than one quarter of the subjects (27.5%) received a loading dose of dydrogesterone (n=2007/7287). Of the subjects who received a loading dose, majority (64%) received 40 mg as loading dose (Table 3). The proportion of patients who received dydrogesterone loading dose for different indications are depicted in (Figure 4). Threatened abortion was the most common indication for which a loading dose was used. Most of the subjects (81.4%) received a 10 mg across indications either as maintenance dose or a regular dose (Table 3). Twice daily (BID) was the most common dosing frequency (66.6%).

#### Concomitant medications received by subjects along with dydrogesterone

The most common concomitant medications being taken by the subjects on along with dydrogesterone included folic acid (45.1%), iron supplements (30.3%) and calcium and vitamin D3 supplements (25.5%). Concomitant medications received by the subjects are captured in (Table 4). Another progesterone preparation (oral, injection, vaginal) other than dydrogesterone was used in 7.8% of subjects.

**Table 4:** Co-prescription medications for subjects prescribed dydrogesterone

Generic name/class of medication	n(%)
Folic acid	3284(45.1)
Iron	2207(30.3)
Calcium ± Vitamin D3 and supplements	1855(25.5)
Other vitamins and multivitamins/antioxidants	808(11.1)
Protein powder	211(2.9)
Thyroid medication	1000(13.7)
Glucose lowering agents	788(10.8)
Antihypertensive	98(1.3)
Progesterone (oral, injection, vaginal, tubal; other than dydrogesterone)	569(7.8)
Estrogen	52(0.7)
HCG	372(5.1)
Myo-inositol (n=11) and other unspecified medications for PCOS related infertility, irregular menstruation	130(1.8)
Unspecified medications for hyperprolactinemia	90(1.2)
Antacids and Anti-acidity	162(2.2)
Medications to control uterine bleeding (Tranexamic acid)	41(0.6)
Antinausea and vomiting in pregnancy	163(2.2)
Aspirin	340(4.7)
Heparin	77(1.1)

## Discussion

Progesterone plays a significant role in the maintenance of a normal menstrual cycle and healthy pregnancy and management of threatened abortion, recurrent pregnancy loss (sequential loss of ≥3 pre-viable pregnancies)^(22)^ and many other obstetrical and gynecological conditions.^(4,23)^ Progesterone is a key management strategy for providing luteal support during IVF and assisted reproductive technology (ART).^(4)^

Progesterone can be administered through several routes such as oral, intramuscular and vaginal.^(4)^ Micronized vaginal progesterone (MVP) capsules can be self-administered, and this is a commonly prescribed, effective and safe method of administering progesterone. However, MVP can cause discomfort due to vaginal irritation and discharge, and may not be culturally acceptable by some women.^(4)^

Oral route of drug administration is the most convenient route for patients.^(4)^ Oral progesterone can be prescribed as micronized progesterone and as dydrogesterone. The Lotus I and II trials showed that oral dydrogesterone can be an effective and safe option to MYP.^(4,24)^ On the other hand, oral micronized progesterone has limited use in clinical practice because it undergoes extensive first-pass metabolism and therefore has low bioavailability.^(4)^ Further, literaure shows that compared to other progesterones, oral micronized progesterone use was associated with the highest risk of miscarriage.^(13)^

A large cross-sectional study from China (N= 91,464) assessing drug utilization patterns in patients with threatened abortion noted that dydrogesterone prescription rates increased over time from 2014 to 2020 while those of other progesterone preparations decreased over time.^(20)^ Dydrogesterone has become the preferred treatment option in progesterone-deficient obstetric and gynecological conditions due to ease of administering an oral dose, similar or better efficacy and tolerability than micronized progesterone, lower dose due to high bioavailability, and improved outcomes in infertility, threatened abortion and recurrent pregnancy loss.^(1,4,5,7,10-13)^

Dydrogesterone has been in use for over 60 years with proven efficacy, safety and tolerability profile across multiple obstetrical and gynecological indications.^(1,7)^ Many large studies with threatened abortion (drug utilization study; China; N= 91,464), recurrent pregnancy loss (systematic review; N=509), for luteal support in IVF (dydrogesterone twice daily vs. micronized vaginal progesterone gel vs. oral micronized capsules; India; N=1,373) or ART (dydrogesterone plus micronized vaginal progesterone gel vs. placebo plus micronized vaginal progesterone gel; India; N=498) show that dydrogesterone is the most commonly used and effective and safe progesterone in these indications.^(7,9,20,25,26)^ However, there are no studies on the utilization pattern of dydrogesterone across various obstetric and gynecological conditions in Indian population. This is a large Indian study, that to best of our knowledge, shows the dydrogesterone utilization patterns in 7287 subjects who routinely received dydrogesterone as part of the SOC for their obstetric or gynecological conditions.

This study reported that threatened abortion and recurrent pregnancy loss were the most common indications for which dydrogesterone was used, followed by recurrent pregnancy loss, infertility due to luteal phase insufficiency and luteal phase support as a part of ART. However, there was great variation in the use of the loading dose, dydrogesterone dosage, dosing frequency, regimen, and duration of dydrogesterone across indications.

The study showed that prior pregnancy loss (27.2%), advanced maternal age (19.2) and obesity (16.2%) were the most common risk factors observed in patients who received dydrogesterone. These are also the most common risk factors identified for threatened and recurrent pregnancy loss, which were the most common indications for which subjects received dydrogesterone in this study.^(27)^ It is important to note that all other risk factors identified (family history, advanced paternal age, associated medical condition, alcohol consumption, defects in the uterine and fallopian tube and smoking) have been associated with any kind of abortion^(27)^ and infertility,^(28)^ the two major indications for prescribing dydrogesterone.

Most of the subjects in this study had a pregnancy complicated by threatened abortion or recurrent pregnancy loss or needed luteal phase support for ART/continuation of pregnancy. In this subject population, PCOS, thyroid disorder, anemia and hypertension were the most commonly observed comorbidities in the subjects of this study. Similar complications were also noted by a large birth cohort study, which reported maternal hypertension, diabetes, thyroid disorders, obesity, asthma, and tobacco use as the most common comorbidities in women prior to and during pregnancy.^(29)^

A loading dydrogesterone dose of 40 mg is usually given for threatened abortion.^(21)^ However, almost half the subjects (48%) with threatened abortion did not receive a loading dose as per the local prescribing information (Chart 1).^(21)^ Similarly, though 40 mg was the most prescribed loading dose (64%), lower loading doses of 20 mg and 30 mg were also used in 19.3% and 15.8% of cases, respectively. Also, loading dose was used for conditions not requiring it as per the local prescribing information such as recurrent pregnancy loss (24%) and infertility due to luteal phase insufficiency (12%).

As per the local prescribing information, dydrogesterone maintenance dose of 20 or 30 mg per day should be prescribed for threatened abortion, while 10 mg to 20 mg should be used across indications that do not require a loading dose (Chart 1). However, this study showed that majority of subjects (81.4%) across indications were on 10 mg dose, used as a maintenance or as continuous/cyclic dose without a loading dose. However, since BID was the most common dosing frequency (66.6%), many patients on 10 mg dose received 20 mg daily, which is in line with the prescribing information.

The study showed that continuous dosing was prescribed in most cases (77.6%). However, dydrogesterone should be prescribed in cyclic dosing for most indications (Chart 1) except in endometriosis and dysfunctional uterine bleeding (DUB) and as continuous sequential therapy with estrogen in hormone replacement therapy (HRT).

The most common concomitant medications being taken by the subjects on dydrogesterone in this study included folic acid (45.1%), iron supplements (30.3%) and calcium and vitamin D3 supplements (25.5%). This is the first study elaborating the concomitant medication used in different obstetrical and gynecological conditions in Indian women who received dydrogesterone.

This study also showed that 7.8% of patients were prescribed multiple progesterone preparations across indications. The cross-sectional study from China too reported that 12% of patients used multiple progesterone preparations for threatened abortion. Of these, dydrogesterone combinations with injectable and oral progesterones were used by 5.6% and 1.1% of patients, respectively. Combination of oral dydrogesterone with another progesterone for luteal support during ART has been found to reduce miscarriage and improve live birth rate.^(30,31)^

The study is limited by its retrospective design and the fact that the accuracy of the data collected depended on the physicians. Further, the study had no control on the indications for which dydrogesterone was prescribed or its dose. In-depth analysis by risk factors, comorbid conditions and concomitant medications used was not performed at the time of writing this manuscript. Also, the frequency of risk factors and comorbid conditions identified are for patients who received dydrogesterone as their SOC. Therefore, the frequency and types of risk factors and comorbid conditions identified cannot be generalized.

However, to the best of our knowledge this is the largest (N=7287) and only study assessing the utilization pattern of dydrogesterone in India. The study covered the indications, risk factors, comorbid conditions and concomitant medication used in patients receiving dydrogesterone. The initial analysis showed the utilization practices of dydrogesterone by indication, dose, dosing frequency and regime. Thus, a clear picture of the patient population that usually receives dydrogesterone emerged and could be useful for the physicians for selecting the right patient for dydrogesterone. The study also helped assess the variations in dydrogesterone utilization and thus helped identify knowledge gaps in drug utilization practices of dydrogesterone.

## Conclusion

The study helped to gain a better understanding of the Indian patient population receiving dydrogesterone in terms of the common clinical indications, frequently prevalent risk factors and comorbid conditions, and the common concomitant medications used in this patient population at real-life scenario.
